# Comparison of Tracheal Diameter Measured by Chest X-Ray and by Computed Tomography

**DOI:** 10.1155/2010/269171

**Published:** 2010-08-19

**Authors:** Shigeki Sakuraba, Ryohei Serita, Junya Kuribayashi, Shizuko Kosugi, Hirofumi Arisaka, Kazuichi Yoshida, Junzo Takeda

**Affiliations:** ^1^Department of Anesthesiology, Clinical Care Medicine, Kanagawa Dental College, Kanagawa 238-8580, Japan; ^2^Department of Anesthesiology, School of Medicine, Keio University, Tokyo 160-8582, Japan; ^3^Department of Anesthesia, Kasumigaura National Hospital, Ibaraki 300-8585, Japan

## Abstract

Assessments of tracheal diameter (TD) are important to select proper endotracheal tubes. Previous studies have used X-ray and physical indices to estimate tracheal diameter but these may not reflect the actual TD. We compared TD measured by X-ray (TD-XP) and by computer tomography (TD-CT) in 200 patients. Also, we analyzed correlation of TD-CT with physical indices such as age, height, weight, and BMI. TD-XP and TD-CT were significantly correlated (male: *n* = 55, *P* = .0146; female: *n* = 91, *P* = .001). TD-XP was 0.4 mm wider in male and 1.0 mm wider in female than TD-CT. However, correlation coefficients of TD-XP and TD-CT are very weak (male: *r* = 0.36; female: *r* = 0.653). TD-CT did not correlate with age, height, weight, or BMI. Our findings suggest that correlations of TD-XP and TD are statistically significant but not clinically significant. Physical indices are not useful to estimate TD.

## 1. Introduction

It is thought to be important to select the appropriate size of endotracheal tube (ETT) to prevent ETT-induced complications, such as airway edema. An overinflated cuff or excessively large ETT relative to tracheal size may induce tracheal mucosal ischemia [1] or hoarseness [2].

Tracheal diameter can generally be measured accurately by CT, but CT images are taken only in a limited number of patients. Chest X-ray images, however, are often taken preoperatively, and used to evaluate the diameter of the trachea so as to determine the tracheal tube size. However, as tracheal diameter measured by X-ray (TD-XP) is not always accurate, in the present study, we compared TD-XP with tracheal diameter measured by CT (TD-CT). Previous studies have attempted to evaluate the correlation of TD-XP with age, height, and weight as a means of making approximate predictions of tracheal diameter. Due to the limited predictive power of TD-XP, we evaluate the correlation of these indices with TD-CT as well.

## 2. Materials and Methods

Following approval by the Kasumigaura National Hospital ethics committee, we conducted a retrospective study using existing data from patients that had undergone cervical vertebrate laminectomy. From February 1999 to December 2001, 200 consecutive patients with cervical spondylosis who received both chest X-ray and CT scanning at the hospital were determined to be eligible for inclusion in the study. Exclusion criteria were used to help minimize statistical bias due to inherited or acquired abnormalities that might cause narrowing of the airway including all of the following: respiratory symptoms or signs (e.g., cough, wheezing, or stridor); a history of sternotomy, which could cause airway deformity due to fibrosis; or opacities in the lungs in either the X-ray or CT images, which could result from airway narrowing due to inflammation. 

In the CT images, the transverse width of the trachea was measured at the level of the VII cervical corpus vertebrae. In the X-ray images, the transverse width was measured at the upper line formed by the connection of the bilateral extremitas sternalis claviculae. Both of these landmarks are thought to correspond to the site at which the cuff of the intubated ETT contacts the mucosa. We refer to these measurements as TD-CT and TD-XP, respectively. 

The Bland-Altman method [3] and linear regression were used to compare TD-CT and TD-XP in each subject. Linear regression was also used to compare TD-CT and each of the following indices: age, height, weight, and BMI. All comparisons were done separately for males and females. A *P* value <.05 was considered significant. Results are expressed as mean ± SD.

## 3. Results

**Figure 1:**
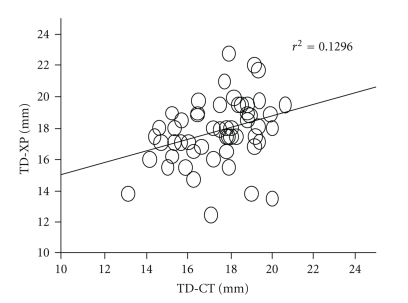
Correlation between TD-XP and TD-CT in male.

**Figure 2:**
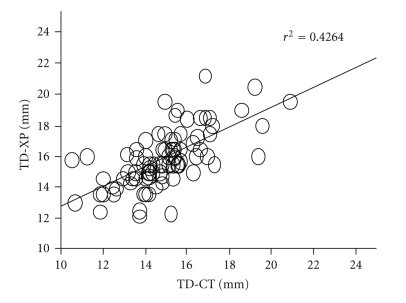
Correlation between TD-XP and TD-CT in female.

**Figure 3:**
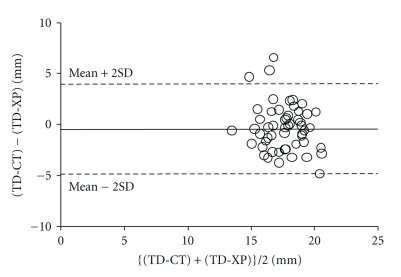
Bland-Altman analysis of TD-CT versus TD-XP in male. The solid line shows bias, and the dotted lines show the limit of agreement.

**Figure 4:**
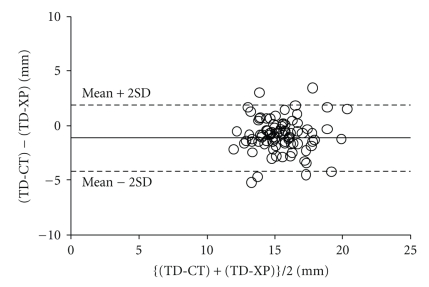
Bland-Altman analysis of TD-CT versus TD-XP in female. The solid line shows bias, and the dotted lines show the limit of agreement.

A total of 146 patients met the inclusion criteria for the study; their demographic and physical characteristics are shown in Table 1. The coefficients of correlation (*r*) between TD-XP and TD-CT were 0.36 in males (*n* = 55, *P* = .0146) (see Figure 1) and 0.653 in females (*n* = 91, *P* = .001) (see Figure 2). Bland-Altman analysis of TD-CT and TD-XP revealed a bias of −0.4 mm with a precision of 2.2 mm in males, and a bias of −1.0 mm with a precision of 1.0 mm in females. The limits of agreement were −4.8 mm/4.0 mm in males (see Figure 3), and −3.0 mm/1.0 mm in females (see Figure 4). 

There was no significant correlation between TD-CT and any of the following indices: age (male: *r* = 0.241, *P* = .765; female: *r* = 0.093, *P* = .3824), height (male: *r* = −0.055, *P* = .693; female: *r* = 0.12, *P* = .256), weight (male: *r* = −0.018, *P* = .897; female: *r* = −0.18, *P* = .866), or BMI (male: *r* = 0.005, *P* = .972; female: *r* = −0.099, *P* = .35).

## 4. Discussion

The measurement of transverse tracheal width is usually performed using noninvasive methods such as chest X-ray, CT [4], MRI [5], or ultrasonography [5, 6]. However, high-quality laryngeal images provided by CT and MRI are not routinely obtained, and the quality of ultrasonography is operator dependent [5]. Furthermore, in adults, neither height nor weight predicts transverse tracheal width [7, 8]. There are no standard formulas for determining proper ETT size. Although not method for measuring transverse tracheal width, the ETT cuff-leak test at an insufflating pressure between 10 and 25 cm H_2_O and an audible air leak check has been proposed as a means for selecting the appropriate ETT size [9, 10]. However, the outer ETT diameter may change in response to pressure, and the audibility of air leaks may vary by physician ability, patient head position, or depth of neuromuscular blockade [9, 10]. Due to these limitations, chest X-ray images are frequently used to measure the transverse tracheal width. To validate this metric, in this study, we evaluated TD-XP by comparison with TD-CT.

However, this study cannot prove any clinically significant correlation of TD-XP with TD-CT. Therefore, we conclude that TD-XP is not useful to select the appropriate size of ETT. Furthermore, TD-XP tends yield overestimates of tracheal diameter.

Also, as past studies shown that TD-XP does not correlate with age, height, or weight [11, 12], we conclude that TD-CT does not correlate with age, height, weight, or BMI.

## Figures and Tables

**Table 1:** Patient characteristics.

	Male (*n* = 55)	Female (*n* = 91)
Age (yr)	58 ± 13 (25–80)	59 ± 15 (16–84)
Height (cm)	164 ± 7 (149–180)	150 ± 7 (136–165)
Weight (kg)	65 ± 11 (49–104)	50 ± 8 (36–76)
BMI (kg m-2)	24.1 ± 3.2 (18.2–35.1)	22.4 ± 3.4 (15.8–33.0)
TD-CT (mm)	17.4 ± 1.7 (13.1–20.6)	14.8 ± 1.8 (10.5–20.9)
TD-XP (mm)	17.7 ± 2.0 (12.5–22.8)	15.8 ± 1.8 (12.1–21.2)

Values are presented as mean ± SD or absolute number (range).

BMI = body mass index.

TD-CT = transverse width of trachea in CT image.

TD-XP = transverse width of trachea in chest X-ray image.
